# Artificial intelligence-assisted accurate diagnosis of anterior cruciate ligament tears using customized CNN and YOLOv9

**DOI:** 10.3389/fradi.2025.1691048

**Published:** 2025-11-04

**Authors:** Taner Alic, Sinan Zehir, Meryem Yalcinkaya, Emre Deniz, Harun Emre Kiran, Onur Afacan

**Affiliations:** ^1^ Orthopaedics and Traumatology Department, Hitit University Faculty of Medicine, Corum, Türkiye; ^2^ Department of Industrial Engineering, Faculty of Engineering, Hitit University, Corum, Türkiye; ^3^ Department of Computer Engineering, Faculty of Engineering, Hitit University, Corum, Türkiye

**Keywords:** anterior cruciate ligament tear, diagnosis, high accuracy, artificial intelligence, deep learning, convolutional neural networks, magnetic resonance imaging

## Abstract

**Background:**

Accurate diagnosis of anterior cruciate ligament (ACL) tears on magnetic resonance imaging (MRI) is critical for timely treatment planning. Deep learning (DL) approaches have shown promise in assisting clinicians, but many prior studies are limited by small datasets, lack of surgical confirmation, or exclusion of partial tears.

**Aim:**

To evaluate the performance of multiple convolutional neural network (CNN) architectures, including a proposed CustomCNN, for ACL tear detection using a surgically validated dataset.

**Methods:**

A total of 8,086 proton density–weighted sagittal knee MRI slices were obtained from patients whose ACL status (intact, partial, or complete tear) was confirmed arthroscopically. Eleven deep learning models, including CustomCNN, DenseNet121, and InceptionResNetV2, were trained and evaluated with strict patient-level separation to avoid data leakage. Model performance was assessed using accuracy, sensitivity, specificity, and area under the receiver operating characteristic curve (AUC).

**Results:**

The CustomCNN model achieved the highest diagnostic performance, with an accuracy of 91.5% (95% CI: 89.5–93.1), sensitivity of 92.4% (95% CI: 90.4–94.2), and an AUC of 0.913. The inclusion of both partial and complete tears enhanced clinical relevance, and patient-level splitting reduced the risk of inflated metrics from correlated slices. Compared with previous reports, the proposed approach demonstrated competitive results while addressing key methodological limitations.

**Conclusion:**

The CustomCNN model enables rapid and reliable detection of ACL tears, including partial lesions, and may serve as a valuable decision-support tool for radiologists and orthopedic surgeons. The use of a surgically validated dataset and rigorous methodology enhances clinical credibility. Future work should expand to multicenter datasets, diverse MRI protocols, and prospective reader studies to establish generalizability and facilitate integration into real-world workflows.

## 1 Introduction

Anterior cruciate ligament (ACL) injuries are among the most common knee pathologies, particularly in athletes and physically active individuals (1). Despite advances in diagnostic imaging techniques and clinical examination methods, misdiagnosis or delayed diagnosis of ACL tears remains a significant clinical challenge (2, 3). Indeed, it has been reported that even experienced orthopedic surgeons may miss the diagnosis at the initial presentation in nearly one-quarter of cases (1–4).

Magnetic resonance imaging (MRI) is considered the most effective noninvasive method for evaluating knee ligament structures, offering a diagnostic accuracy of over 90%. However, factors such as hemarthrosis, synovial effusion, or low-contrast images can complicate the detailed assessment of ligamentous structures and may lead to false-negative results (5, 6).

In recent years, artificial intelligence (AI) applications, particularly deep learning approaches, have been increasingly integrated into musculoskeletal radiology. Convolutional neural networks (CNN), capable of automatic feature extraction and classification, have shown promising results in detecting various knee pathologies, including cartilage lesions, meniscal tears, and ligament injuries (7, 8).

Nevertheless, the number of CNN-based models specifically optimized for detecting ACL tears on MRI using large-scale clinical datasets remains limited. Therefore, in this study, we developed a two-step deep learning approach to identify ACL tears on knee MRI scans. In the first step, anatomical region localization was performed using a YOLOv9-based segmentation model. Subsequently, eleven different CNN architectures, including a newly designed customized CNN (CustomCNN) model, were comparatively evaluated.

The aim of our study is to demonstrate the potential of these models to enhance diagnostic accuracy and to support clinicians in decision-making processes in orthopedic practice.

## 2 Methodology

**Figure 1:**
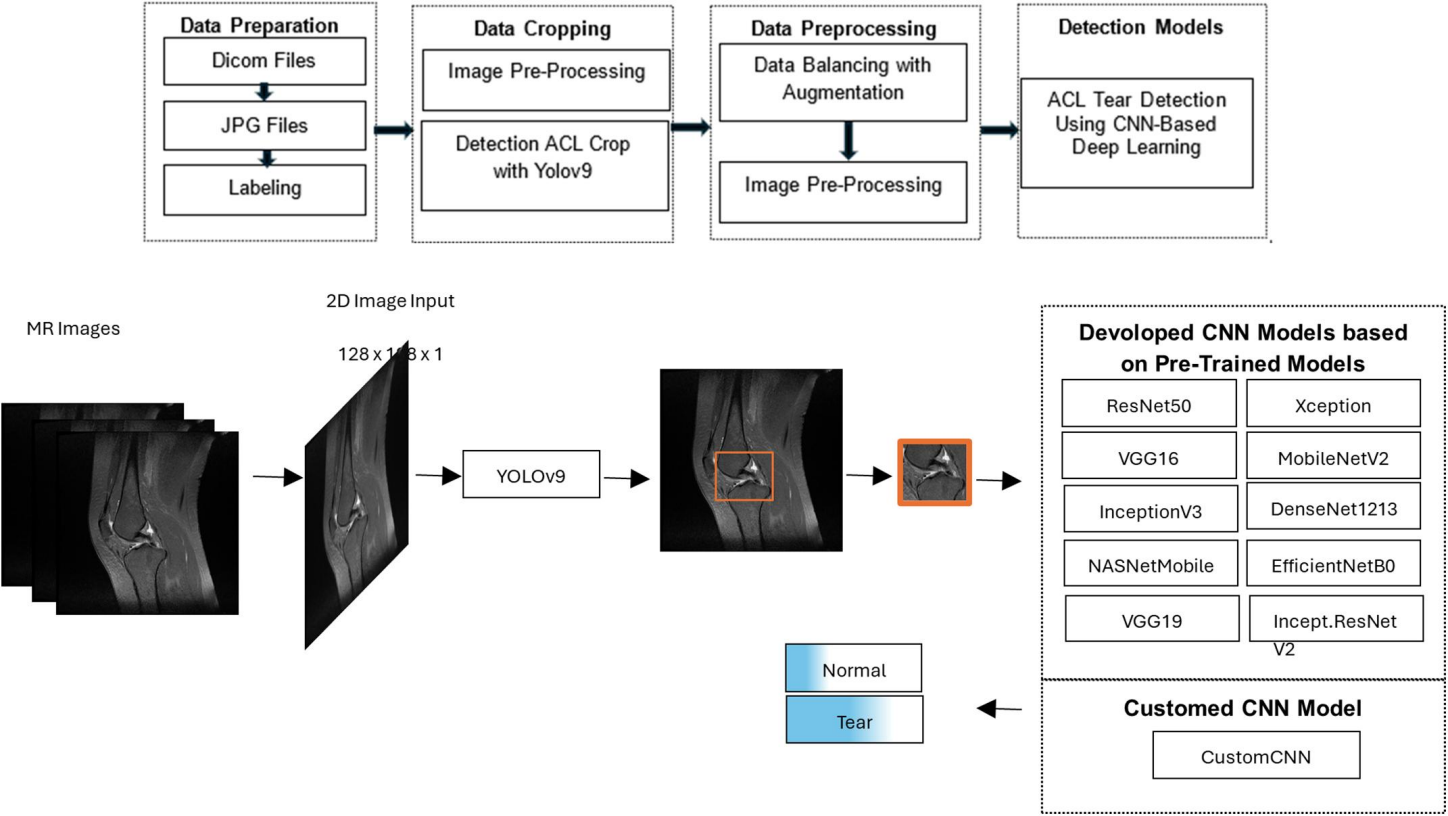
Workflow of the proposed deep learning model for detecting anterior cruciate ligament tears on knee MRI, consisting of a YOLOv9-based segmentation step to localize and crop the ligament region, followed by classification of the cropped images as torn or intact using CNN models.

In this study, a two-stage deep learning approach was developed for the detection of ACL tears in knee MRI, as illustrated in Figure 1. In the first stage, the region of interest (ROI) containing the ACL was automatically localized within the knee anatomy using a YOLOv9-based image segmentation model. These localized areas were then cropped from the original images, enabling the model to focus only on clinically relevant regions.

### 2.1 The dataset and image preparation

This retrospective study received institutional IRB/ethics approval (University Ethics Committee, 2023), after which knee MRI examinations performed between March 2016 and December 2023 were reviewed in the hospital's internal database to compile the dataset for the deep learning study.

A total of 253 patients were included, of whom 122 had arthroscopically confirmed complete or partial ACL tears and 131 had no ACL pathology. In this study, complete and partial tears were not treated as separate classes; both were categorized as “tear,” as partial tears often require similar management to complete tears. From these patients, 8,086 sagittal PD-weighted MRI slices were obtained. After YOLOv9-based cropping, 1,230 representative slices were retained (836 torn, 394 normal).

Because the number of normal slices was lower than torn slices, class balancing was performed at the image-slice level. To this end, data augmentation was applied only to the training set, including:

• Random rotation within ±20°
• Horizontal flipping
• Brightness adjustment (80%–100%)
• Minor scale/zoom transformations (0.9–1.1)

These augmentations increased the number of normal slices from 394 to 844, yielding a balanced dataset of 1,680 slices (844 normal, 836 torn). No augmentation or artificial balancing was applied to validation or test sets.

Each MR image was converted from DICOM format to JPEG using Python (version 3.11.5) and the pydicom library (version 2.4.4). Labeling was performed using LabelImg for use in YOLOv9 and CNN architectures.

### 2.2 Detection ACL cropping with YOLOv9

In the preprocessing phase of the YOLOv9 model, the Contrast Limited Adaptive Histogram Equalization (CLAHE) method was used to enhance image contrast, followed by hyperparameter optimization during YOLOv9 tuning. The final image was obtained through masking and weighting processes using Gaussian and median blur filters to soften edges and reduce noise.

The dataset was divided into five folds for cross-validation and trained over five iterations, each consisting of 200 epochs, a batch size of 16, and a resolution of 640 × 640 pixels. Ground-truth bounding-box annotations for segmentation were manually created by two orthopedic surgeons using LabelImg and verified by a third researcher (inter-annotator agreement ≈95%).

Segmentation performance was quantified using precision, recall, and IoU-thresholded mean Average Precision (mAP). YOLOv9 achieved precision = 0.999, recall = 1.000, mAP@0.5 = 0.995, and mAP@0.5:0.95 = 0.581. The lower mAP@0.5:0.95 value is expected under stricter IoU thresholds and reflects variability across multiple overlap criteria, particularly for small, elongated structures such as the ACL region. In addition, segmentation was further evaluated using Intersection over Union (IoU) and Dice coefficient metrics, achieving an average IoU of 0.86 and Dice coefficient of 0.91 across validation images. These findings confirm robust localization performance and support reliable ACL segmentation.

The 1,230 cropped sequences were divided into a training set (1,202 examinations, 189 patients), tuning set (236 examinations, 31 patients), and validation set (242 examinations, 32 patients). Stratified random sampling ensured each set contained 50% positive and 50% negative examples, enhancing generalization and minimizing false positive and negative rates.

### 2.3 ACL tear detection using CNN-based deep learning

Cropped images from YOLOv9 were processed through the following preprocessing pipeline: CLAHE (clip limit = 8.0, tile grid size = 4 × 4) → Niblack thresholding (75%) → resizing to 128 × 128 pixels → normalization to 1/255.

To detect ACL tears, additional layers were integrated into pre-trained CNN architectures (ResNet50, VGG16, InceptionV3, NASNetMobile, Xception, MobileNetV2, DenseNet121, EfficientNetB0, Inception-ResNetV2), while a CustomCNN architecture was independently developed. Balanced data was used for all models. Training and validation images were randomly adjusted in brightness (80%–100%) during training. Model parameters with the lowest validation loss were saved. All computations were performed on a desktop equipped with an Intel Core i7-13700F CPU, 32 GB RAM, and Nvidia GeForce RTX 4090 GPU with 24 GB GDDR6X RAM.

### 2.4 Custom CNN model (CustomCNN)

The CustomCNN model was specifically designed to efficiently extract hierarchical features from ACL MRI slices:

• The first 3 × 3 convolutional layer with 64 filters captures fine-grained local patterns.
• The subsequent 5 × 5 convolutional layers with 128 and 256 filters capture broader spatial relationships, helping to identify ACL tear patterns of varying shapes and sizes.
• The final 3 × 3 layers with 512 and 256 filters consolidate extracted features and enhance generalization before the fully connected layers.

Each convolutional layer is followed by Batch Normalization and ReLU activation, with Max Pooling and Dropout layers to improve training stability and prevent overfitting. The flattened output passes through two fully connected layers (128 → 256 neurons, both with BatchNorm, ReLU, and Dropout), and a final softmax output layer produces the classification results.

This architecture balances accuracy with computational efficiency and was empirically found to outperform simpler or deeper variants on our dataset.

### 2.5 Developed CNN models based on pre-trained models

Pre-trained CNN models (VGG16, ResNet50, InceptionV3, Xception, DenseNet121, EfficientNetB0, MobileNetV2, NASNetMobile, Inception-ResNetV2, VGG19) were customized by adding Batch Normalization, Dense, and Dropout layers, followed by softmax activation. Most layers were frozen; only the last layers were trainable.

All classifiers were trained on 2D grayscale ROIs (128 × 128) cropped by YOLOv9; no 3D volumes were used. Training employed batch size = 10, 60 epochs, Adam optimizer (lr = 1 × 10⁻⁴), and binary cross-entropy. Performance was assessed using accuracy, precision, recall, F1-score, and AUC. Data splitting was patient-level with 70%/15%/15% train/tuning/test ratio. Stratified sampling preserved 1:1 class balance in tuning/test subsets. Data augmentation (brightness 0.8–1.0, horizontal flip, minor affine transforms) was applied only to the training set; validation/test sets were not augmented.

## 3 Results

### 3.1 Segmentation results

The YOLOv9 model demonstrated excellent performance in localizing the ACL region. As summarized in Table 1, segmentation achieved a precision of 0.999, recall of 1.000, and a mean Average Precision (mAP) of 0.995 at an IoU threshold of 0.5. The stricter mAP@0.5:0.95 metric yielded 0.581, which is consistent with expected variability across multiple IoU thresholds, particularly for small and elongated structures such as the ACL. These findings confirm that the ROI cropping provided by YOLOv9 was reliable and minimized the risk of downstream classification errors attributable to poor localization.

**Table 1:** Performance metrics of YOLOv9 model for ACL region segmentation.

Metric	Value
Precision	0.999
Recall (Sensitivity)	1.000
IoU-based mAP @0.5	0.995
IoU-based mAP @0.5:0.95	0.581

### 3.2 Classification results

A total of 11 deep learning models were trained and evaluated. To ensure robustness, test results were repeated 100 times, and median values with 95% confidence intervals were reported (Table 2).

**Table 2:** Performance metrics of eleven deep learning models for anterior cruciate ligament (ACL) tear detection, including accuracy, precision, recall, F1 score, and AUC.

Model	Accuracy	Precision	Recall	F1 Score	AUC
CustomCNN	0.915 (0.895,0.931)	0.924 (0.899, 0.944)	0.924 (0.904, 0.942)	0.924 (0.907, 0.939)	0.913 (0.893, 0.93)
DenseNet121	0.905 (0.889, 0.93)	0.914 (0.897, 0.935)	0.919 (0.895, 0.951)	0.916 (0.901, 0.937)	0.903 (0.886, 0.929)
InceptionResNetV2	0.899 (0.879,0.915)	0.91 (0.888, 0.934)	0.907 (0.886, 0.93)	0.909 (0.893, 0.925)	0.896 (0.877, 0.915)
EfficientNetB0	0.889 (0.869,0.907)	0.897 (0.876, 0.92)	0.907 (0.884, 0.93)	0.902 (0.885, 0.917)	0.886 (0.867, 0.905)
MobileNetV2	0.886 (0.868,0.905)	0.907 (0.88, 0.932)	0.89 (0.857, 0.916)	0.898 (0.881, 0.914)	0.886 (0.865, 0.907)
VGG16	0.882 (0.859,0.904)	0.861 (0.841, 0.885)	0.942 (0.919, 0.965)	0.9 (0.881, 0.918)	0.874 (0.849, 0.895)
ResNet50	0.876 (0.861,0.895)	0.906 (0.888, 0.927)	0.872 (0.846, 0.895)	0.888 (0.873, 0.905)	0.878 (0.861, 0.897)
NASNetMobile	0.873 (0.846,0.896)	0.852 (0.821, 0.88)	0.936 (0.919, 0.959)	0.891 (0.872, 0.911)	0.863 (0.835, 0.888)
Xception	0.856 (0.833,0.878)	0.821 (0.796, 0.841)	0.953 (0.93, 0.977)	0.882 (0.865, 0.899)	0.842 (0.818, 0.865)
InceptionV3	0.846 (0.824,0.868)	0.817 (0.789, 0.843)	0.936 (0.913, 0.959)	0.874 (0.855, 0.891)	0.834 (0.808, 0.857)
VGG19	0.824 (0.804,0.846)	0.811 (0.787, 0.829)	0.895 (0.872, 0.93)	0.852 (0.833, 0.87)	0.813 (0.794, 0.836)

Among all models, CustomCNN exhibited the highest performance, achieving 91.5% accuracy, 92.4% sensitivity, 92.4% precision, and an AUC of 0.913. DenseNet121 and InceptionResNetV2 also demonstrated high accuracy and sensitivity, with AUCs of 0.903 and 0.896, respectively. EfficientNetB0 achieved 88.9% accuracy and 90.7% sensitivity. Other models performed moderately, with AUC values ranging from 0.824 to 0.873.

The preprocessing steps, including patient-level splitting, YOLOv9-based ROI cropping, and data balancing through augmentation, contributed to these high performance metrics by enhancing generalizability and reducing potential bias. These results confirm that the proposed framework provides reliable detection of ACL tears and highlights the benefit of integrating segmentation and classification pipelines.

Table 3 summarizes the performance of our CustomCNN model in comparison with recent state-of-the-art (SOTA) studies published between 2022 and 2025. Despite some studies reporting higher accuracy numerically, our model demonstrates competitive performance with 91.5% accuracy, 92.4% sensitivity, and an AUC of 0.913, while benefiting from arthroscopically confirmed labels, inclusion of both complete and partial ACL tears, patient-level data separation to prevent leakage, and rigorous 100-run evaluation with confidence intervals. This highlights the clinical reliability and robustness of the proposed CustomCNN framework.

**Table 3:** Comparison of CustomCNN performance with recent SOTA studies (2022–2025).

Study	Year	Model	Dataset (n)	Accuracy	Sensitivity/Recall	AUC
Abdullah et al. (15)	2022	BP-ANN	90 patients	94.4%	0.88	–
Bien et al. (9)	2023	CNN-based AI	NR	NR	0.76	–
Chang et al. (11)	2024	Customized 3D CNN	4,144 MRIs	>96%	NR	–
Liu et al. (12)	2025	Automatic CNN	350 MRIs	NR	0.96	0.98
CustomCNN (our study)	2025	CustomCNN	8,086 MRIs	91.5%	92.4%	0.913

## 4 Discussion

In this study, we investigated the applicability and performance of various customized deep learning architectures for the evaluation of ACL tears. Eleven deep learning models were assessed and the three top-performing architectures were CustomCNN, DenseNet121, and InceptionResNetV2. The CustomCNN model achieved the highest performance, with an accuracy of 91.5% (95% CI: 89.5–93.1) and a sensitivity of 92.4% (95% CI: 90.4–94.2); its discriminative ability was further supported by an area under the receiver operating characteristic curve (AUC) of 0.913. Importantly, ACL region localization using the YOLOv9 segmentation model provided highly accurate ROI crops (precision = 0.999; recall = 1.000; mAP@0.5 = 0.995; mAP@0.5:0.95 = 0.581), ensuring that downstream classifiers received focused, clinically relevant image patches.

The performance of our CustomCNN model was also compared with recent SOTA studies published between 2022 and 2025 (Table 3). Despite some models reporting higher accuracy in different datasets, our framework demonstrates competitive performance with 91.5% accuracy and 92.4% sensitivity, while using arthroscopically validated labels, including both partial and complete ACL tears, and enforcing patient-level separation to prevent data leakage. This comparison highlights that the proposed CustomCNN provides reliable and clinically relevant ACL tear detection comparable to recent SOTA works.

Compared to similar conditions reported in the literature, our study demonstrates competitive performance and high accuracy. Many prior deep learning studies on ACL tears, such as those by Bien et al. (9) and Tran et al. (10), relied solely on radiological reports for labeling, without surgical confirmation, which limits reliability. In contrast, all labels in our dataset were validated arthroscopically, thereby reducing misclassification risk and enhancing diagnostic credibility. Furthermore, unlike most earlier works that focused only on complete tears, our dataset included both complete and partial ACL tears to better reflect real-world clinical practice. However, we did not stratify torn cases by subtype, which prevents assessment of model performance for partial vs. complete tears specifically. This distinction, together with our patient-level data splitting, provides stronger clinical validity but also highlights the need for larger multicenter studies to further establish generalizability.

While studies by Chang et al. (11) and Liu et al. (12) focused exclusively on complete ACL tears, partial tears were often ignored or considered intact, which can lead to diagnostic misinterpretation and artificially inflated accuracy rates. In our study, both complete and partial tears were included within the torn category to better reflect real-world clinical practice, thereby increasing clinical relevance. However, torn cases were not stratified into partial vs. complete subgroups, and we therefore cannot provide subtype-specific performance. Future studies should adopt a three-class labeling approach (intact, partial, complete) with sufficient sample sizes to separately evaluate these clinically distinct entities and to better inform surgical treatment planning.

Liu et al. (12) developed an automatic system using CNNs to isolate ACL and detect structural abnormalities on a sagittal PD-weighted dataset of 350 MRIs, achieving 96% sensitivity and specificity (AUC 0.98). In comparison, our study analyzed a substantially larger dataset of 8,086 sagittal PD-weighted knee MRI sequences. A critical methodological distinction was that all data splits were performed at the patient level, ensuring that no images from the same individual appeared in more than one subset. This strategy prevented potential data leakage and inflated performance estimates that can arise from correlated slices. Such rigorous patient-level separation is rarely considered in prior AI studies, many of which rely heavily on data augmentation without accounting for intra-patient correlations. Moreover, our results were based on 100 experimental repetitions with median values and 95% confidence intervals reported, further enhancing the robustness and reproducibility of the findings.

Deep learning-based approaches support early and accurate ACL tear diagnosis on MRI images, providing significant assistance to clinical experts (9, 13, 14). These methods enable rapid and accurate assessment of larger patient groups, offering time- and cost-effectiveness. In our pipeline, the integration of YOLOv9 segmentation ensured accurate localization of the ACL region prior to classification, reducing irrelevant image context and potentially improving sensitivity for subtle lesions. However, we did not perform a direct comparison of classification performance using manual (ground-truth) crops vs. YOLOv9-based crops; therefore, the residual influence of segmentation errors on classifier accuracy remains a limitation that should be addressed in future studies. AI-assisted diagnostic systems should ultimately be integrated into clinical decision-making as supportive tools that complement, rather than replace, the expertise of radiologists and orthopedic surgeons.

This study has several limitations. First, the dataset size should be expanded, and multicenter data are needed to validate model performance across diverse demographics, MRI vendors, and acquisition protocols. Although fat-suppressed sagittal sequences were chosen to enhance ligament and soft tissue visibility, performance may differ on coronal or axial planes or on non–fat-suppressed images. Future evaluations using multiple imaging protocols would therefore be beneficial. Second, although both partial and complete ACL tears were included, we did not stratify tears by subtype, which prevents reporting of differential performance for partial vs. complete lesions. Third, class balancing was achieved partly through augmentation, which cannot fully substitute for naturally balanced datasets and may introduce bias. Fourth, while YOLOv9 demonstrated excellent segmentation accuracy, we did not compare classification performance using manual (ground-truth) vs. automated crops, leaving the residual effect of segmentation errors unquantified. Finally, the model has not yet been externally validated, and a prospective reader study integrating AI outputs with radiologist interpretation will be an essential step toward clinical deployment.

In conclusion, this study developed and evaluated an artificial intelligence-based framework for ACL tear detection using a clinically validated dataset of 8,086 knee MRI slices. Among the 11 tested deep learning architectures, the CustomCNN model achieved the best diagnostic performance, with 91.5% accuracy, 92.4% sensitivity, and an AUC of 0.913. Methodological innovations—including patient-level data separation and the inclusion of both complete and partial tears—enhanced clinical validity and minimized bias, distinguishing our work from many previous reports. While segmentation using YOLOv9 was highly accurate, we did not compare classifier performance with ground-truth manual crops, which remains a limitation. Larger multicenter studies incorporating diverse MRI protocols, vendors, and demographics are required to confirm generalizability. Ultimately, AI-assisted diagnostic systems such as ours hold promise as supportive tools to accelerate image interpretation, guide clinical decision-making, and improve patient outcomes when integrated responsibly into orthopedic practice.

## 5 Conclusions

In conclusion, this study developed and evaluated an artificial intelligence-based framework for ACL tear detection using a clinically validated dataset of 8,086 knee MRI slices. Among the 11 tested deep learning architectures, the CustomCNN model achieved the best diagnostic performance, with 91.5% accuracy, 92.4% sensitivity, and an AUC of 0.913. Methodological innovations—including patient-level data separation and the inclusion of both complete and partial tears—enhanced clinical validity and minimized bias, distinguishing our work from many previous reports. While segmentation using YOLOv9 was highly accurate, we did not compare classifier performance with ground-truth manual crops, which remains a limitation. Larger multicenter studies incorporating diverse MRI protocols, vendors, and demographics are required to confirm generalizability. Ultimately, AI-assisted diagnostic systems such as ours hold promise as supportive tools to accelerate image interpretation, guide clinical decision-making, and improve patient outcomes when integrated responsibly into orthopedic practice.

## Data Availability

The raw data supporting the conclusions of this article will be made available by the authors, without undue reservation.
